# CHK1 plays a critical role in the anti-leukemic activity of the wee1 inhibitor MK-1775 in acute myeloid leukemia cells

**DOI:** 10.1186/s13045-014-0053-9

**Published:** 2014-08-01

**Authors:** Wenxiu Qi, Chengzhi Xie, Chunhuai Li, J Timothy Caldwell, Holly Edwards, Jeffrey W Taub, Yue Wang, Hai Lin, Yubin Ge

**Affiliations:** 1 National Engineering Laboratory for AIDS Vaccine, Key Laboratory for Molecular Enzymology & Engineering, the Ministry of Education, and School of Life Sciences, Jilin University, Changchun, China; 2 Department of Oncology, Wayne State University School of Medicine, 110 East Warren Ave, Detroit, 48201, MI, USA; 3 Molecular Therapeutics Program, Barbara Ann Karmanos Cancer Institute, Wayne State University School of Medicine, 110 East Warren Ave, Detroit, MI, USA; 4 Department of Pediatric Hematology and Oncology, The First Hospital of Jilin University, Changchun, China; 5 MD/PhD Program, Wayne State University School of Medicine, 540 E. Canfield Ave, Detroit, MI, USA; 6 Cancer Biology Program, Wayne State University School of Medicine, 110 East Warren Ave, Detroit, MI, USA; 7 Department of Pediatrics, Wayne State University School of Medicine, 540 E. Canfield Ave, Detroit, MI, USA; 8 Division of Pediatric Hematology/Oncology, Children’s Hospital of Michigan, 3901 Beaubien Blvd, Detroit, MI, USA; 9 Department of Hematology and Oncology, The First Hospital of Jilin University, Changchun, China

**Keywords:** Wee1, MK-1775, CHK1, Acute myeloid leukemia

## Abstract

**Background:**

Acute myeloid leukemia (AML) remains a difficult disease to treat and requires new therapies to improve treatment outcome. Wee1 inhibitors have been used to prevent activation of the G2 cell cycle checkpoint, thus enhancing the antitumor activity of DNA damaging agents. In this study, we investigated MK-1775 in AML cell lines and diagnostic blast samples to identify sensitive subtypes as well as possible mechanisms of resistance.

**Methods:**

In vitro MK-1775 cytotoxicities of AML cell lines and diagnostic blasts were measured using MTT assays. The effects of MK-1775 on cell cycle progression and related proteins were determined by propidium iodide (PI) staining and flow cytometry analysis and Western blotting. Drug-induced apoptosis was determined using annexin V/PI staining and flow cytometry analysis.

**Results:**

We found that newly diagnosed and relapsed patient samples were equally sensitive to MK-1775. In addition, patient samples harboring t(15;17) translocation were significantly more sensitive to MK-1775 than non-t(15;17) samples. MK-1775 induced apoptosis in both AML cell lines and diagnostic blast samples, accompanied by decreased phosphorylation of CDK1 and CDK2 on Tyr-15 and increased DNA double-strand breaks (DSBs). Time-course experiments, using AML cell lines, revealed a time-dependent increase in DNA DSBs, activation of CHK1 and subsequent apoptosis following MK-1775 treatment, which could be attenuated by a CDK1/2 inhibitor, Roscovitine. Simultaneous inhibition of CHK1 and Wee1 resulted in synergistic anti-leukemic activity in both AML cell lines and primary patient samples *ex vivo*.

**Conclusions:**

Our study provides compelling evidence that CHK1 plays a critical role in the anti-leukemic activity of MK-1775 and highlights a possible mechanism of resistance to MK-1775. In addition, our study strongly supports the use of MK-1775 to treat both newly diagnosed and relapsed AML, especially cases with t(15;17) translocation, and supports the development of combination therapies with CHK1 inhibitors.

## Introduction

Acute myeloid leukemia (AML) is a challenging disease to treat, with overall survival rates of 65% for children and 25% for adults [1],[2]. Resistance to cytarabine (ara-C) and anthracycline [e.g. daunorubicin (DNR)]-based chemotherapy remains a major cause of treatment failure. Therefore, new therapies are greatly needed to treat this disease.

One mechanism of chemotherapy resistance is the induction of G2/M cell cycle arrest, which allows cells to repair and survive DNA damage [3]. Wee1 kinase is a cell cycle checkpoint protein whose primary function is inhibitory phosphorylation of CDK1 and CDK2 on Tyr-15, preventing progression through G2/M and S phase, respectively [4],[5]. Inhibitory phosphorylation of CDK1 and CDK2 on Tyr-15 is removed by CDC25 phosphatases [6],[7]. CDC25 activity is inhibited when phosphorylated by CHK1, which is in turn controlled primarily by ATR kinase [8],[9]. Upon sensing DNA damage or replication stress ATR phosphorylates CHK1, activating it and eventually leading to S and G2 cell cycle arrest, allowing for DNA repair [8],[9].

The first selective and potent Wee1 inhibitor, MK-1775, has been primarily used to target the G2 checkpoint to exert toxicity in cells with impaired p53 function [10]. It has been demonstrated that when combined with DNA damaging agents it is able to abrogate the G2 checkpoint and enhance apoptosis [10]-[18]. In addition, Wee1 inhibition has been shown to induce DNA damage through the induction of replication stress secondary to overactive CDKs and inhibition of DNA repair [19].

In this study, we used AML cell lines and blast samples obtained either at diagnosis or at relapse to investigate the cytotoxic effects of MK-1775 on AML. Our results suggest that MK-1775 may be equally effective in newly diagnosed and relapsed AML. Additionally, we demonstrate that patient samples harboring t(15;17) translocation are significantly more sensitive to MK-1775 than others. Furthermore, our study suggests that MK-1775 treatment induces DNA damage which activates CHK1, CHK1 phosphorylates CDC25, inhibiting the dephosphorylation of CDK1/2, thus countering the effects of MK-1775. Activation of CHK1 can be overcome by the addition of a CHK1 inhibitor, resulting in synergistic anti-leukemic activity. This demonstrates a potential mechanism of resistance to MK-1775 treatment and highlights the importance of combination therapies.

## Results

### MK-1775 induces DNA damage and apoptosis in AML cells

**Figure 1:**
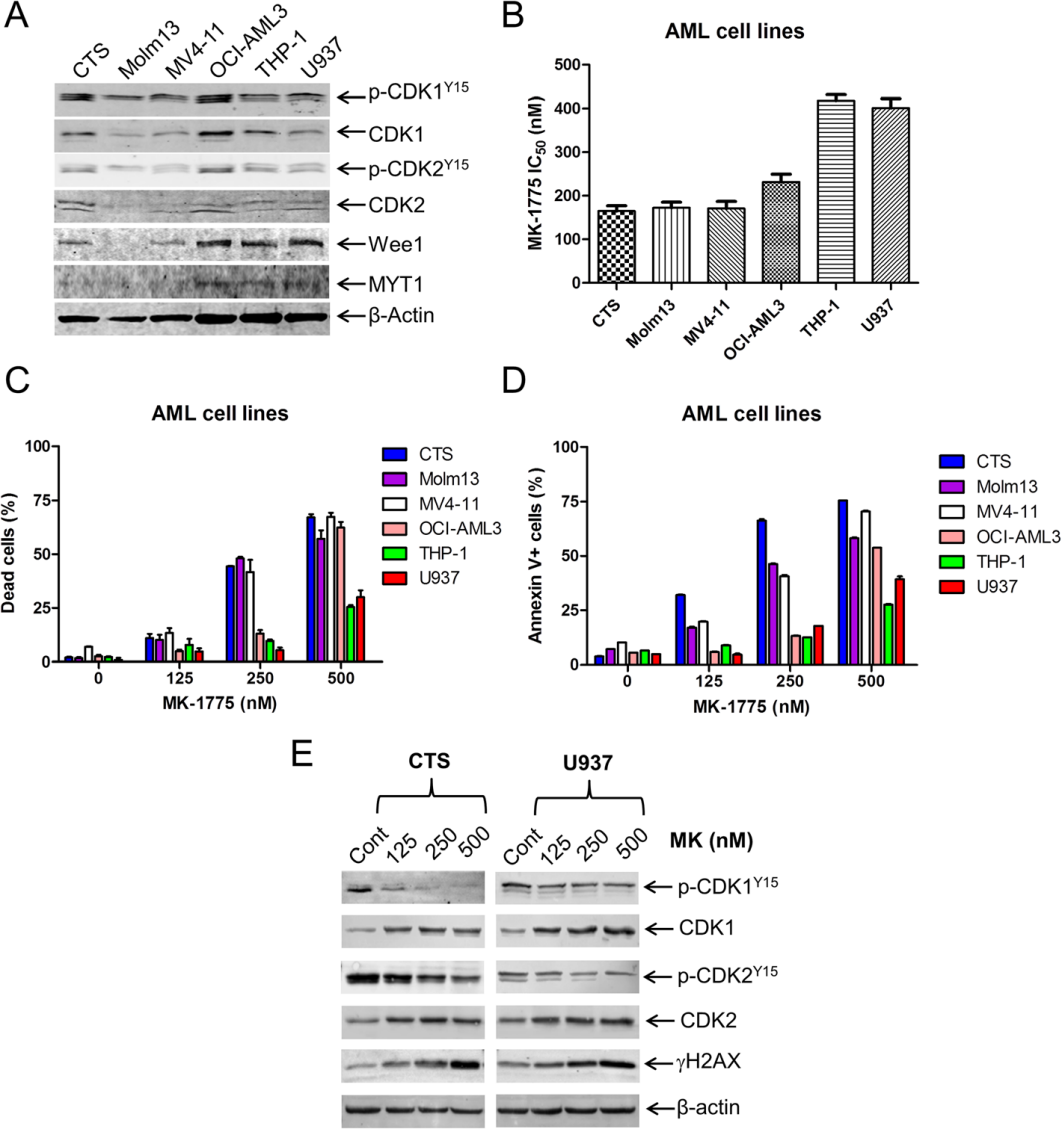
**MK-1775 induces apoptosis in AML cells.** Panel **A**: Protein extracts from 6 AML cell lines were subjected to Western blotting and probed with anti-Wee1, -Myt1, -p-CDK1 (Y15), -CDK1, -p-CDK2 (Y15), -CDK2, or -β-actin antibody. Panel **B**: AML cell lines were cultured for 72 h in complete medium with variable concentrations of MK-1775 (MK) and viable cell numbers were determined using MTT assays. IC_50_ values were calculated as drug concentration necessary to inhibit 50% growth compared to untreated control cells. The data for panels **B** and **C** are presented as mean values ± standard errors from at least 3 independent experiments. Panel **C**-**E**: AML cells were treated with MK-1775 for 48 h. The percentage of dead cells was determined by Trypan blue exclusion **(Panel C)**. Apoptotic events were determined by annexin V/PI staining and flow cytometry analyses **(Panel D)**. The data for panel **D** are presented as mean values ± standard errors of triplicates from one representative experiment which was repeated 3 independent times. Whole cell lysates were subjected to Western blotting, and probed with anti-p-CDK1, -CDK1, -p-CDK2, -CDK2, -γH2AX, or -β-actin antibody **(Panel E)**. Representative Western blots are shown.

To investigate the effects of MK-1775 in AML cells, first we determined the protein levels of Wee1, p-CDK1, CDK1, p-CDK2, CDK2 and MYT1 in six AML cell lines. The proteins were expressed at variable levels (Figure 1A). Next, we tested drug sensitivity by MTT assays. The IC_50_s ranged from about 200-400 nM after 72 h treatment (Figure 1B). To determine if MK-1775 induces cell death, we treated the cell lines with up to 500 nM MK-1775 for 48 h and assessed viability using the trypan blue exclusion assay. As shown in Figure 1C, there was a concentration-dependent increase in dead cells for all six cell lines tested. Annexin V/PI staining and flow cytometry analysis revealed that MK-1775 caused concentration-dependent increase in apoptotic cells (Figure 1D). This was accompanied by decreased p-CDK1 and p-CDK2 and increased total CDK1 and CDK2 (Figure 1E). There was also a concentration-dependent increase of phosphorylated H2AX (γH2AX), indicating increased DNA double-strand breaks (DSBs) [γH2AX is an established biomarker for DNA DSBs [20]]. The CTS and U937 cell lines were chosen as representative AML cell lines for further mechanistic studies.

**Figure 2:**
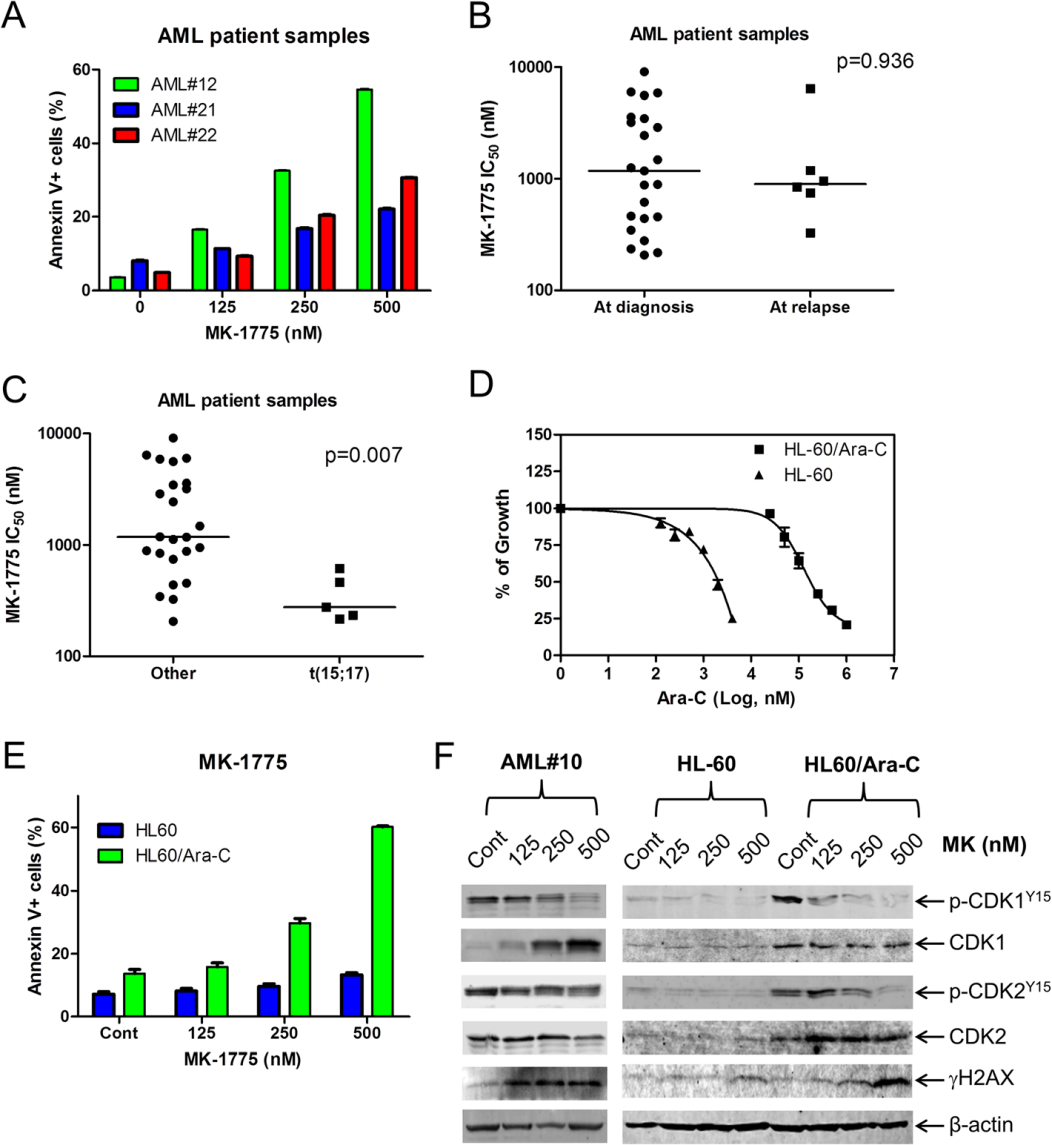
**Diagnostic AML blasts from patients either at first diagnosis or at relapse are equally sensitive to MK-1775.** Panel **A**: Freshly isolated AML patient samples were purified by standard Ficoll-Hypaque density centrifugation then treated with MK-1775 for 48 h and apoptotic events were determined by annexin V/PI staining and flow cytometry analyses. Panels **B** and **C**: *Ex vivo* MK-1775 (MK) sensitivity was determined using MTT assays. The horizontal lines indicate median MK-1775 IC_50_s in each group of AML patient samples. Panel **D**: Cytarabine sensitivity was determined using MTT assays for the HL-60 and HL-60/Ara-C cell lines. Panel **E**: The HL60 and HL60/Ara-C cells were treated with MK-1775 for 48 h and apoptotic events were determined by annexin V/PI staining and flow cytometry analyses. The data are presented as mean of triplicates ± standard errors from one representative experiment. Panel **F**: Freshly isolated cells from patient AML#10 were purified by standard Ficoll-Hypaque density centrifugation. AML#10, HL-60, and HL-60/Ara-C cells were treated with MK-1775 for 48 h. Whole cell lysates were subjected to Western blotting and probed with anti-p-CDK1, -CDK1, -p-CDK2, -CDK2, -γH2AX, or -β-actin antibody.

Next, we determined *ex vivo* MK-1775 sensitivity in freshly isolated AML blast samples (n = 29). MK-1775 IC_50_s ranged from 217 nM (AML#16) to 6.4 μM (AML#27, Table 1). Similar to the cell lines, we detected a concentration-dependent increase in apoptosis for three patient samples after MK-1775 treatment (Figure 2A). Interestingly, the median MK-1775 IC_50_ for the diagnostic (n = 23) and relapse samples (n = 6) were similar (1176 and 896.1 nM, respectively, p = 0.936, Figure 2B). In addition, we found that patient samples harboring t(15;17) translocation (n = 5) were significantly more sensitive to MK-1775 than non-t(15;17) samples (n = 24, p = 0.007, Figure 2C). Next, we generated a cytarabine resistant cell line (HL60/Ara-C) to determine if they would exhibit cross-resistance to MK-1775. Despite being approximately 600-times more resistant to cytarabine than the parental HL-60 cells (Figure 2D), HL-60/Ara-C cells were more sensitive to MK-1775 treatment (Figure 2E). There was a concentration-dependent increase in apoptotic cells for the HL60/Ara-C cell line, whereas the parental cell line remained relatively unaffected by MK-1775 concentrations up to 500 nM. A concentration-dependent decrease in p-CDK1 and p-CDK2 accompanied by increase of γH2AX was detected in cells from patient AML#10 as well as in HL60/Ara-C (Figure 2F). HL60 cells treated with 500 nM MK-1775 had a small increase of γH2AX and no change in p-CDK1 or p-CDK2, probably due to very low levels of expression prior to drug treatment.

**Table 1:** Patient characteristics and MK-1775 sensitivity for primary AML patient samples

**Patient**	**Gender**	**Age (year)**	**Disease status**	**FAB subtype**	**WBC (×10**^**3**^**/μL)**	**Blast purity (%)**	**Cytogenetics**	**MK-1775 IC**_**50**_**(nM)**
AML#0	Female	13	At diagnosis	M2	39.9	79	46, XX, t(8;21)(q22;q22)	5996.5
AML#1	Female	50	At diagnosis	M4	22.66	52	46, XX, del(5q), add(12p)	2448.5
AML#2	Male	20	At diagnosis	M2	23.55	42	45, X, -Y, t(8;21)(q22;q22)	3204.0
AML#3	Male	4	At diagnosis	M3	4.71	75	46, XY, t(15;17)(q22;q21)	461.0
AML#4	Male	55	At relapse	M2	5.64	14	46, XY, t(8;21)(q22;q22)	844.0
AML#5	Female	57	At relapse	M2	22.16	70	46, XX	948.3
AML#6	Male	60	At diagnosis	M3	1.83	86	46, XY, t(15;17)(q22;q21)	233.7
AML#7	Female	53	At relapse	M4	3.87	36	46, XX	325.0
AML#8	Male	55	At diagnosis	M2	3.87	32	46, XY	887.9
AML#9	Female	35	At relapse	M5	17.71	87	47, XX, +10, t(16;21)(p11;q22), add(11p)	744.4
AML#10	Male	23	At diagnosis	M2	25.89	53	46, XY, del(9q)	439.7
AML#11	Female	43	At diagnosis	M2	25.12	91	46, XX	411.3
AML#12	Male	6	At diagnosis	M3	93.99	85	46, XY, t(15;17)(q22;q21)	277.5
AML#13	Male	52	At diagnosis	M2	1.65	27	46, XY, t(11;15;17)(q25;q15;q21)	3579.5
AML#14	Female	18	At diagnosis	M3	1.62	64	46, XX	9113
AML#15	Female	63	At diagnosis	M2	0.6	23	46, XX	344.1
AML#16	Male	38	At diagnosis	M3	2.27	90	46, XY, t(15;17)(q22;q21)	216.9
AML#17	Female	45	At diagnosis	M4	1.52	36	46, XX	3473.0
AML#18	Female	19	At relapse	M2	21.55	52	46, XX, t(8;21)(q22;q22)	1187.0
AML#19	Male	4	At diagnosis	M4	77.32	41	46, XY	5587.0
AML#21	Male	42	At diagnosis	M4	10.60	51	46, XY/44, XY, -17, -19, (11q-?)	1176.0
AML#22	Female	47	At diagnosis	M5	244.53	80	46, XX	1255.0
AML#23	Male	60	At diagnosis	M5	2.10	81	46, XY, +2, +8, I(12)(q10)	1480.0
AML#25	Female	12	At diagnosis	M3	5.11	88	46, XX, t(15;17)(q22;q21)	615.1
AML#26	Male	59	At diagnosis	M2	129.98	81	46, XY	5868.0
AML#27	Female	41	At relapse	M4	40.20	24	47, XX,del(5q),+8,t(15;18)(q12,q23)	6384.0
AML#29	Male	9	At diagnosis	M4	68.14	31	46, XY, t(6;9)(p22;q34)	880.4
AML#31	Male	17	At diagnosis	M2	12.94	69	46, XY	453.7
AML#32	Female	76	At diagnosis	M5	13.93	85	46, XX	2888.0

**Figure 3:**
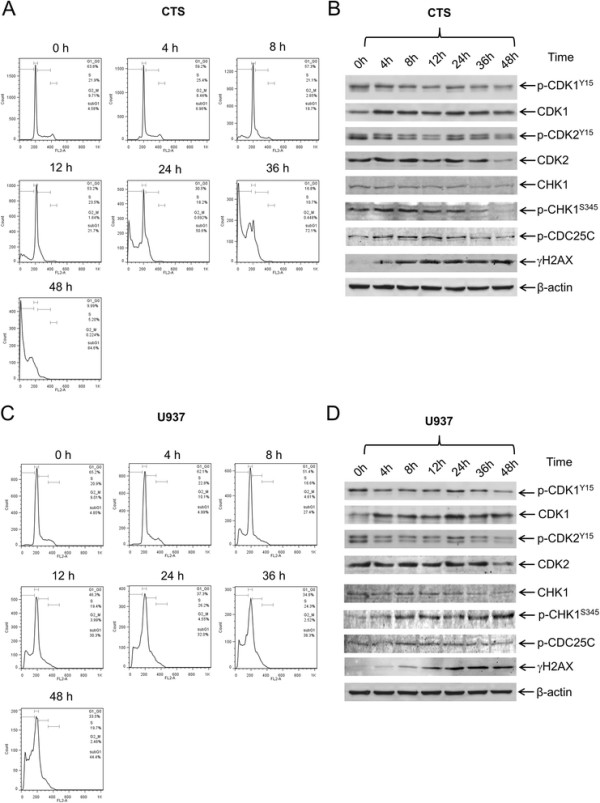
**MK-1775 treatment abrogates the G2/M cell cycle checkpoint.** CTS cells **(Panels A and B)** or U937 cells **(Panels C and D)** were treated with 500 nM MK-1775 for up to 48 h. Samples were taken at the indicated time points and fixed with ethanol for PI staining and cell cycle analysis **(Panels A and C)**. Whole cell lysates were subjected to Western blotting and probed with anti-p-CDK1, -CDK1, -p-CDK2, -CDK2, -CHK1, -pCHK1, -p-cdc25c, -γH2AX, or -β-actin antibody **(Panels B and D)**.

Next, we investigated the effects of MK-1775 on cell cycle progression in both CTS and U937 cells. Treatment with MK-1775 for 48 h revealed a concentration-dependent decrease of the G2/M population accompanied by concentration-dependent increase of the sub-G1 population (Additional file 1: Figure S1). Time course experiments revealed a time-dependent increase of the sub-G1 population and abrogation of the G2 checkpoint for both cell lines (Figure 3A&C and Additional file 2: Table S1). These changes were accompanied by a time-dependent increase of γH2AX as well as a decrease of p-CDK1 and p-CDK2 (Figure 3B&D). Increased total CDK1 levels were detected following MK-1775 treatment. Increased p-CHK1 was detected as early as 4 h following MK-1775 treatment.

### CDK activity is required for MK-1775 anti-leukemic activity

**Figure 4:**
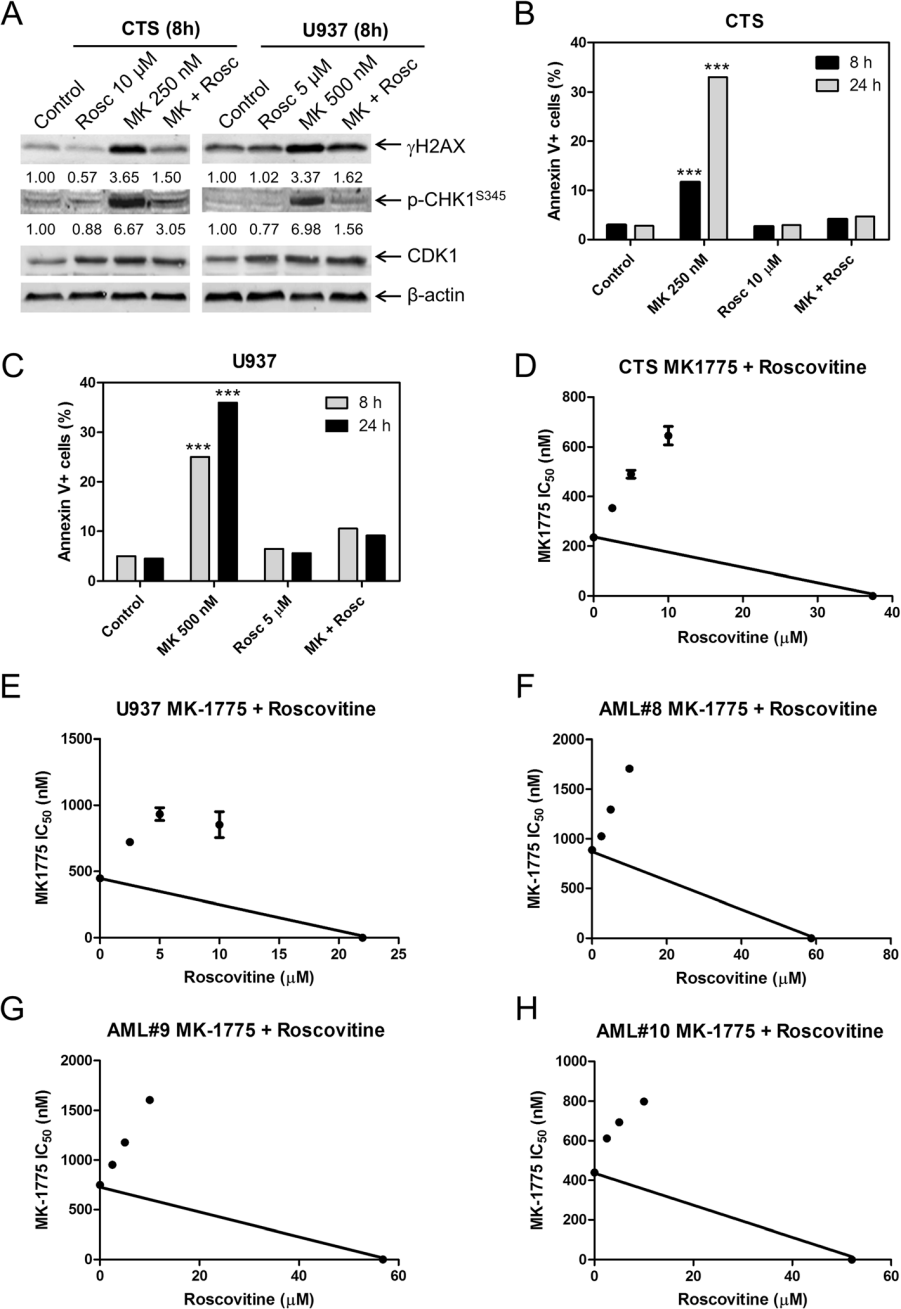
**MK-1775-induced DNA damage and apoptosis are dependent on CDK activity.** Panel **A**: U937 and CTS cell lines were treated for 8 h with the indicated concentrations of Roscovitine (Rosc) and MK-1775. Whole cell lysates were subjected to Western blotting and probed with anti-γH2AX, -p-CHK1, -CDK1, or -β-actin antibody. Densitometry measurements, as described in the Materials and methods section, are shown below the corresponding Western blot. Panels **B** and **C**: CTS and U937 cell lines were treated with MK-1775 and Roscovitine, alone or in combination, for 8 h and 24 h. Apoptosis was measured by annexin V/PI staining and flow cytometry analyses. The data are presented as mean of triplicates ± standard error from one representative experiment. ***indicates p < 0.0005. Panels **D**-**H**: Standard isobologram analyses of anti-leukemic interactions between MK-1775 and Roscovitine in the CTS cell line **(Panel D)**, U937 cell line **(Panel E)**, and patient samples **(Panels F-H)**. The IC_50_ values of each drug are plotted on the axes; the solid line represents the additive effect, while the points represent the concentrations of each drug resulting in 50% inhibition of proliferation. Points falling below the line indicate synergism whereas those above the line indicate antagonism. The data for the CTS and U937 cell lines are presented as mean values ± standard errors from at least three independent experiments, while the data for the patient samples are presented as mean values of duplicates from one experiment.

To determine if CDK activity is required for MK-1775 induced DNA damage and apoptosis, we treated AML cells with Roscovitine, a CDK inhibitor. There was a concentration-dependent decrease in viable cells after Roscovitine treatment for both CTS and U937 cells, as measured by MTT assays (Additional file 1: Figure S2A). Increased γH2AX and p-CHK1 was observed following 8 h MK-1775 treatment, which was substantially abolished by the addition of Roscovitine (Figure 4A and Additional file 1: Figure S3A). MK-1775 induced apoptosis at both 8 h and 24 h in both CTS and U937 cell lines. Combined MK-1775 and Roscovitine treatment abolished MK-1775 induced apoptosis (Figure 4B&C). MTT assays revealed clearly antagonistic anti-leukemic interactions as shown in the standard isobolograms (Figure 4D&E). Similar results were obtained with primary AML patient samples (Figure 4F-H). These results demonstrate that CDK activity is required for MK-1775 anti-leukemic activity in AML cells.

### CHK1 inhibitor LY2603618 synergizes with MK-1775 to induce apoptosis in AML cells

**Figure 5:**
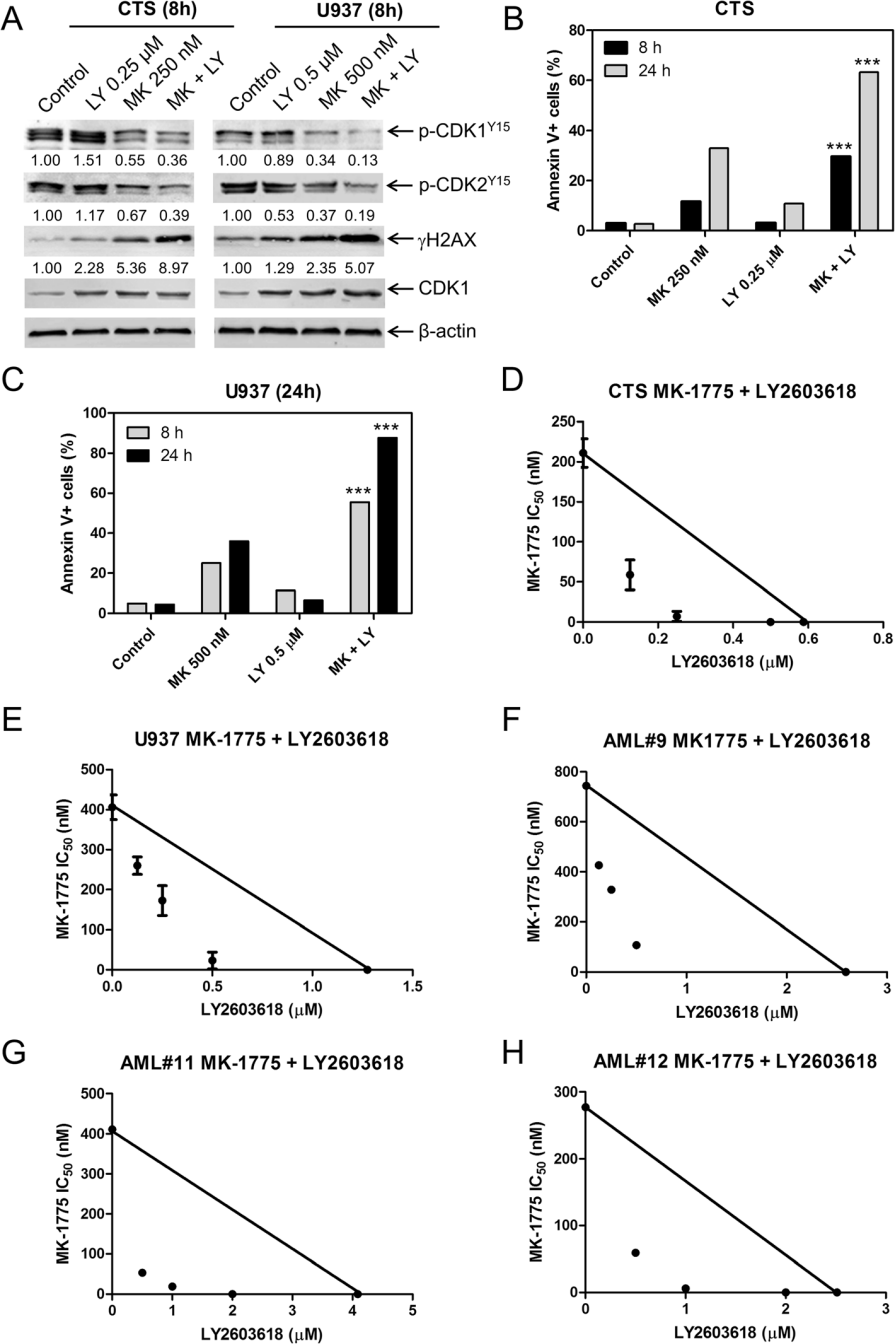
**MK-1775 and LY2603618 synergize to induce apoptosis in AML cell lines and primary patient samples.** Panel **A**: U937 and CTS cells were treated for 8 h. Whole cell lysates were subjected to Western blotting and probed with anti-γH2AX, -pCHK1, -p-cdc25c, -p-CDK1, -p-CDK2, -CDK1, or -β-actin antibody. Densitometry measurements, as described in the Materials and methods section, are shown below the corresponding Western blot. Panels **B** and **C**: CTS and U937 cells were treated with MK-1775 and LY2603618, alone or in combination, for 8 h and 24 h. Apoptotic events were determined by annexin V/PI staining and flow cytometry analyses. The data are presented as mean of triplicates ± standard error from one representative experiment. ***indicates p < 0.0005. Panels **D**-**H**: Standard isobologram analyses of antitumor interactions between MK-1775 and LY2603618 in the CTS cell line **(Panel D)**, U937 cell line **(Panel E)**, and patient samples **(Panels F-H)**. The IC_50_ values of each drug are plotted on the axes; the solid line represents the additive effect, while the points represent the concentrations of each drug resulting in 50% inhibition of proliferation. Points falling below the line indicate synergism whereas those above the line indicate antagonism. The data for the CTS and U937 cell lines are presented as mean values ± standard errors from at least three independent experiments, while the data for the patient samples are presented as mean of duplicates from one experiment.

CTS and U937 cells were treated with MK-1775 plus the selective CHK1 inhibitor LY2603618 for 8 h. There was a concentration-dependent decrease in viable cells following LY2603618 treatment for both CTS and U937 cells (Additional file 1: Figure S2B). γH2AX levels were increased after MK-1775 treatment, which was further increased by the addition of LY2603618 (Figure 5A and Additional file 1: Figure S3B). CDK1 and CDK2 phosphorylation was lower after MK-1775 treatment compared to vehicle control and was further decreased in the combined MK-1775 and LY2603618 treatment. MK-1775-induced apoptosis was significantly enhanced by the addition of LY2603618 (Figure 5B&C). MTT assays revealed synergistic anti-leukemic interactions from the combined MK-1775 and LY2603618 treatment as displayed by the standard isobolograms (Figure 5D&E). Similar results were obtained with primary AML patient samples (Figure 5F-H).

## Discussion

Wee1 inhibitors have primarily been used to target the G2 cell cycle checkpoint, which is activated in response to DNA damaging agents, and has shown promising results when combined with standard chemotherapy drugs [10]-[18]. Using single agent treatment we demonstrate that AML patient samples with t(15;17) translocation are more sensitive to MK-1775 than non-t(15;17), suggesting a possible treatment for patients with t(15;17) who do not respond to all-*trans* retinoic acid and/or arsenic trioxide based therapies. The t(15;17) translocation results in rearrangement of the promyelocytic leukemia (*PML*) gene and the retinoic acid receptor α (*RARα*) gene, generating the PML/RARα fusion protein [21]. This fusion protein acts as a strong transcriptional repressor for numerous genes which are involved in proliferation, DNA repair, and cell death [22],[23], and thus may contribute to the elevated MK-1775 sensitivity. We also found that newly diagnosed and relapsed AML patient samples have similar *ex vivo* MK-1775 sensitivities, as measured by MTT assays. In addition, our studies in a cytarabine resistant cell line further support the preclinical development of MK-1775 for the treatment of relapsed AML.

In our study, we demonstrate that MK-1775 treatment results in increased phosphorylation of CHK1 and H2AX, in agreement with Chaudhuri et al.’s report [13]. Furthermore, increased phosphorylation of both CHK1 and H2AX is dependent on CDK activity. Inhibition of CHK1 in combination with MK-1775 resulted in synergistic anti-leukemic activities in both AML cell lines and primary patient samples. Although combined Wee1 and CHK1 inhibition has been reported in various cancer types [24]-[26], including AML [13], our study confirmed the enhanced anti-leukemic activity with a different CHK1 inhibitor, LY2603618, than has been previously reported (MK-8776, PF-00477736, AR458323) and found similar synergistic anti-leukemic activity. Thus, adding further evidence for the clinical efficacy of combined Wee1 and CHK1 inhibition for the treatment of AML.

**Figure 6:**
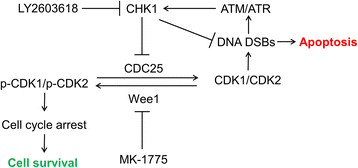
**Proposed mechanisms for the anti-leukemic interaction of MK-1775 and LY2603618 in AML cells.** MK-1775 inhibits Wee1, leading to decreased inhibitory phosphorylation of CDK1/2, allowing CDK1/CDK2 to remain active. This eventually leads to DNA DSBs which triggers activation of ATM/ATR and activates CHK1. Active CHK1 inhibits CDC25s leading to decreased removal of the inhibitory phosphorylation on CDK1/2, thus limiting the amount of active CDK1/2 and the resulting DNA damage from MK-1775 treatment. The addition of a CHK1 inhibitor (e.g. LY2603618) would inhibit the CHK1 DNA repair pathway, allowing for the DNA damage to accumulate and cause apoptosis.

Based on our own results and those previously reported, we propose a mechanism for the cooperative anti-leukemic activity of MK-1775 and LY2603618 in Figure 6. MK-1775 inhibits Wee1, inhibiting phosphorylation of CDK1/2, thus allowing CDK1/CDK2 to remain active. Overactive CDK1/2 eventually leads to DNA DSBs triggering activation of ATM/ATR and CHK1. Activated CHK1 phosphorylates CDC25s, leading to decreased removal of the inhibitory phosphorylation on CDK1/2, in turn limiting the amount of active CDK1/2, arresting the cell cycle to allow for adequate DNA damage repair. The addition of LY2603618 inhibits the inhibitory phosphorylation of CDC25s, accordingly maintaining their phosphatase activity, which maintains active CDK1/2 pools, perpetuating the DNA damage induced by MK-1775 treatment and eventually resulting in enhanced apoptosis.

In summary, our study demonstrates that MK-1775 has potential as an anti-leukemic drug in the treatment of AML. Particularly, our patient sample data supports the investigation of MK-1775 in relapsed AML, as they had similar sensitivities as newly diagnosed cases. In addition, AML cases with t(15;17) translocation were significantly more sensitive to MK-1775 treatment, suggesting a possible role for patients who do not respond to all-*trans* retinoic acid and/or arsenic trioxide-based therapies. We also demonstrate that MK-1775 in combination with cytarabine results in synergistic anti-leukemic activity in AML cell lines and patient samples (Additional file 1: Figure S4). Lastly, our results highlight CHK1 activation as a potential mechanism of resistance to MK-1775 treatment, thus supports the development of combination therapies.

## Materials and methods

### Drugs

MK-1775 (MK), Roscovitine (Rosc), and LY2603618 (LY) were purchased from Selleck Chemicals (Houston, TX, USA). Cytarabine (ara-C) was purchased from Sigma-Aldrich (St. Louis, MO, USA).

### Cell culture

The THP-1, MV4-11, U937, HL-60 cell lines were purchased from the American Type Culture Collection (Manassas, VA, USA). The OCI-AML3 cell line was purchased from the German Collection of Microorganisms and Cell Cultures (DSMZ, Braunschweig, Germany). MOLM-13 cells were purchased from AddexBio (San Diego, CA, USA). The CTS cell line was a gift from Dr. A Fuse from the National Institute of Infectious Diseases, Tokyo, Japan. The cell lines were cultured in RPMI 1640 (except OCI-AML3, which was cultured in alpha-MEM) with 10-15% fetal bovine serum (Hyclone, Logan, UT, USA), 2 mM L-glutamine, 100 U/ml penicillin and 100 μg/ml streptomycin. All cells were cultured in a 37°C humidified atmosphere containing 5% CO2/95% air.

AML blast samples obtained either at diagnosis or at relapse were purified by standard Ficoll-Hypaque density centrifugation, then cultured in RPMI 1640 with 20% fetal bovine serum supplemented with ITS solution (Sigma-Aldrich) and 20% supernatant of the 5637 bladder cancer cell line [as a source of granulocyte-macrophage colony-stimulating factor [27],[28]].

HL-60 cytarabine resistant cells (designated HL-60/Ara-C) were generated by stepwise selection of HL-60 cells in the presence of cytarabine, until they could be maintained in the presence of 600 nM cytarabine.

### Clinical samples

Diagnostic blast samples were obtained from the First Hospital of Jilin University. Written informed consent was provided according to the Declaration of Helsinki. This study was approved by the Human Ethics Committee of the First Hospital of Jilin University. Clinical samples were screened for FLT3-ITD, NPM1, C-kit, CEBPA, IDH1, IDH2 and DNMT3A gene mutations. The samples were also screened for the following fusion genes by real-time RT-PCR: PML-RARα, BCR-ABL, AML1-MDS1, MLL-AF10, MLL-AF4, MLL-ELL, SET-CAN, TLS-ERG, NPM-RARα, E2A-PBX1, AML1-EAP, MLL-AF17, MLL-AF6, MLL-ENL, SIL-TAL1, HOX11, PLZF-RARα, TEL-AML1, DEK-CAN, MLL-AF1p, MLL-AF9, NPM-ALK, TEL-ABL, EIP1L1-PDGFRA, AML1-ETO, CBFB-MYH11, E2A-HLF, MLL-AF1q, MLL-AFX, NPM-MLF1, dupMLL, and TEL-PDGFB.

### In vitro cytotoxicity assays

*In vitro* cytotoxicities of the AML cells were measured by using MTT (3-[4,5-dimethyl-thiazol-2-yl]-2,5-diphenyltetrazoliumbromide, Sigma-Aldrich) assays, as previously described [29],[30]. Briefly, the cells were treated with variable concentrations of MK-1775 for 72 hours. MTT was added to a final concentration of 1 mM and cells were incubated for 4 hours at 37°C. The cells were lysed overnight using 10% SDS in 10 mM HCL and plates were read at 590 nm using a microplate reader. IC_50_ values were calculated as drug concentrations necessary to inhibit 50% growth compared to vehicle control treated cells. The cell line IC_50_ values are presented as mean values ± standard errors from at least three independent experiments. The IC_50_ values for the patient samples are means of duplicates from one experiment, due to limited sample. Patient samples for the combined drug treatments were chosen based on sample availability.

### Western blot analysis

Cells were lysed in the presence of protease and phosphatase inhibitors (Roche Diagnostics, Indianapolis, IN, USA). Whole cell lysates were subjected to SDS-polyacrylamide gel electrophoresis, electrophoretically transferred onto polyvinylidene difluoride (PVDF) membranes (Thermo Fisher Inc., Rockford, IL, USA) and immunoblotted with anti-p-CDK1 (Y15), -CDK1, -p-CDK2 (Y15), -CDK2, -Wee1, -MYT1, -γH2AX, -CHK1, -p-CHK1 (S345), -p-CDC25C (S216) (Cell Signaling Technology, Danvers, MA, USA), or -β-actin (Sigma-Aldrich) antibody, as previously described [31],[32]. Immunoreactive proteins were visualized using the Odyssey Infrared Imaging System (Li-Cor, Lincoln, NE, USA), as described by the manufacturer. Western blots were repeated at least three times and one representative blot is shown. Only one patient sample was used for MK-1775 treatment and subsequent Western blot analysis due to the limited amount of sample available. Densitometry measurements were made using Odyssey V3.0 (Li-Cor), normalized to β-actin, and then fold change relative to no drug control was calculated.

### Apoptosis

AML cells were treated with MK-1775, Roscovitine, LY2603618, or the indicated combinations and subjected to flow cytometry analysis to determine drug-induced apoptosis using an annexin V-fluorescein isothiocyanate (FITC)/propidium iodide (PI) apoptosis Kit (Beckman Coulter; Brea, CA, USA), as previously described [29],[33]. Results are expressed as percent of annexin V + cells. Experiments with AML cell lines were performed 3 independent times in triplicates and data presented are from one representative experiment, while patient sample experiments were performed once in triplicates. Data are presented as mean values ± standard errors from one representative experiment. Due to limited sample, only three patient samples were evaluated for MK-1775-induced apoptosis by flow cytometry.

### Cell cycle progression

Cells were treated with the indicated drugs for up to 48 h. The cells were harvested and fixed with ice-cold 80% (v/v) ethanol for 24 h. The cells were pelleted, washed with PBS, and resuspended in PBS containing 50 μg/mL propidium iodide (PI), 0.1% Triton X-100 (v/v), and 1 μg/mL DNase-free RNase. DNA content was determined by flow cytometry analysis using a FACScan flow cytometer (Becton Dickinson, San Jose, CA, USA) as previously described [34]. Cell cycle analysis was performed using Multicycle software (Phoenix Flow Systems, Inc., San Diego, CA, USA). Histograms were created using FlowJo v7.6.5 (Tree Star, Ashland, OR, USA).

### Statistical analysis

Differences in cell apoptosis between treated (individually or combined) and untreated cells were compared using the pair-wise two-sample *t*-test. The *p* value for the differences between MK-1775 IC_50_s for the groups of patient samples was calculated using the Mann–Whitney *U* test. Statistical analyses were performed with GraphPad Prism 5.0.

## Competing interest

The authors declare that they have no competing interests.

## Authors’ contributions

WQ and CX performed the molecular biology studies. CL, JWT, YW, HL, and YG participated in the design and coordination of the study. CL, JTC, HE, JWT, YW, HL, and YG participated in the data analysis and interpretation. CL, JTC, HE, JWT, YW, HL, and YG helped to draft the manuscript. All authors read and approved the final manuscript.

## Additional files

## Supplementary Material

Additional file 1: Figure S1. MK-1775 causes concentration-dependent abrogation of the G2/M cell cycle checkpoint. **Figure S2.** Concentration-dependent decrease in viable cells after roscovitine or LY2603618 treatment. **Figure S3.** Densitometry measurements for the western blot experiments corresponding to Figures 4A and 5A. **Figure S4.** MK-1775 and cytarabine synergize in AML cells. Click here for file

Additional file 2: Table S1. Cell cycle distribution of CTS and U937 cells following MK-1775 treatment. Click here for file
