# Supplementary material for: CHK1 plays a critical role in the anti-leukemic activity of the wee1 inhibitor MK-1775 in acute myeloid leukemia cells
Source: J Hematol Oncol. 2014 Aug 1;7:53. doi: 10.1186/s13045-014-0053-9 (PMC4237862; doi:10.1186/s13045-014-0053-9)
Supplement: Additional file 1: Figure S1. — MK-1775 causes concentration-dependent abrogation of the G2/M cell cycle checkpoint. Figure S2. Concentration-dependent decrease in viable cells after roscovitine or LY2603618 treatment. Figure S3. Densitometry measurements for the western blot experiments corresponding to Figures 4A and 5A. Figure S4. MK-1775 and cytarabine synergize in AML cells. [file s13045-014-0053-9-S1.pdf]

**ADDITIONAL FILE 1**

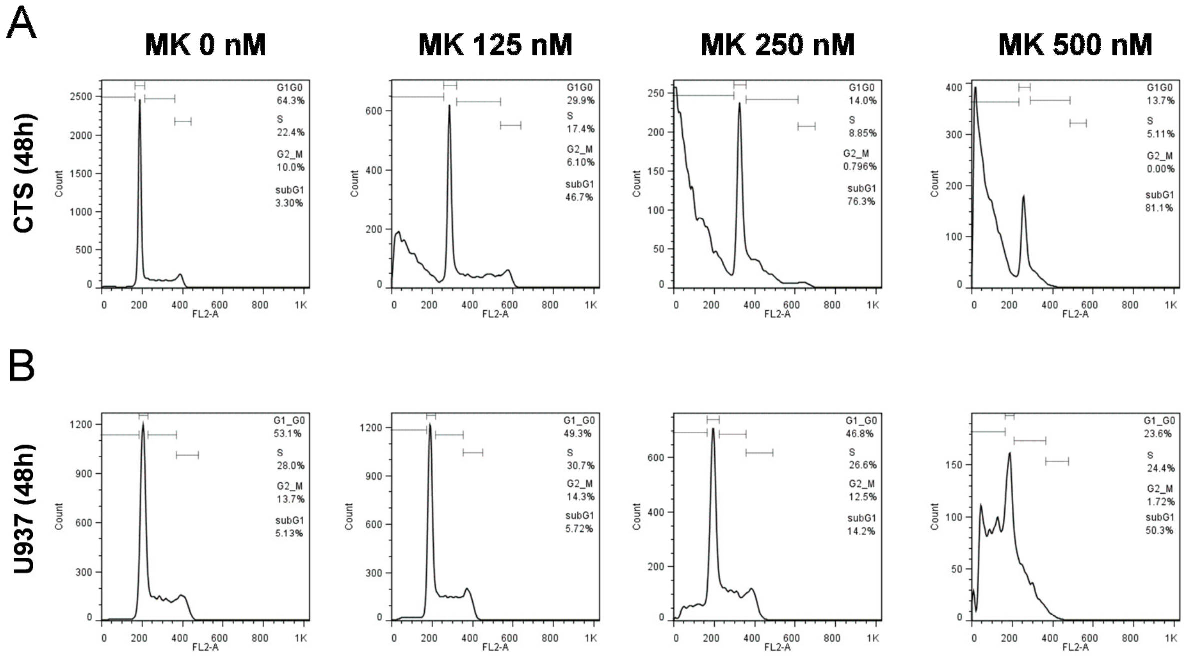


**Figure S1.** MK-1775 causes concentration-dependent abrogation of the G2/M cell cycle checkpoint. CTS (**panel A**) or U937 (**panel B**) cells were treated for 48 h with varying concentrations of MK-1775. Samples were fixed with ethanol for PI staining and cell cycle analysis.

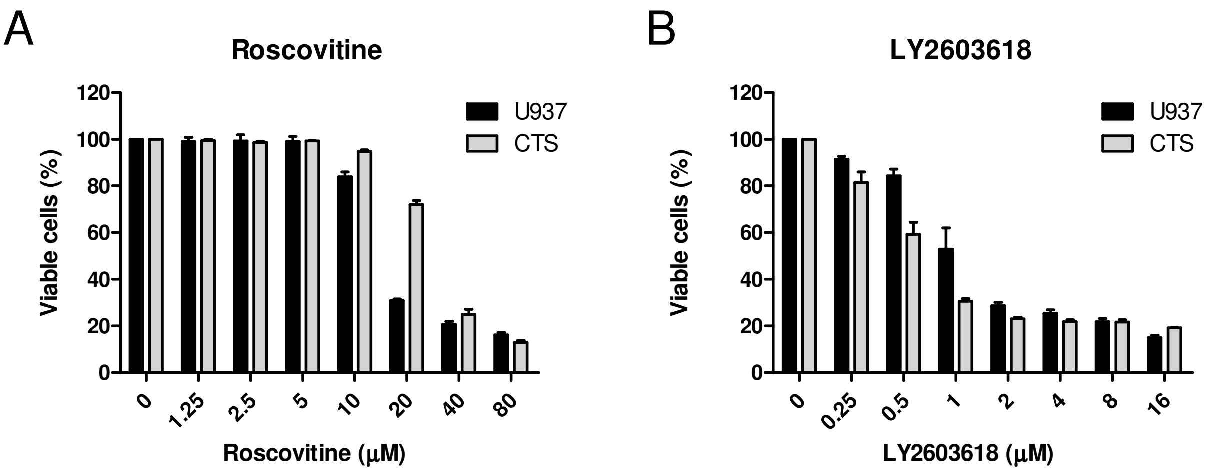


**Figure S2.** Concentration-dependent decrease in viable cells after roscovitine or LY2603618 treatment. **Panel A:** CTS and U937 cells were treated with 0-80 µM roscovitine for 72 h and viable cell numbers were determined using MTT assays. **Panel B:** CTS and U937 cells were treated with 0-16 µM LY2603618 for 72 h and viable cell numbers were determined using MTT assays.

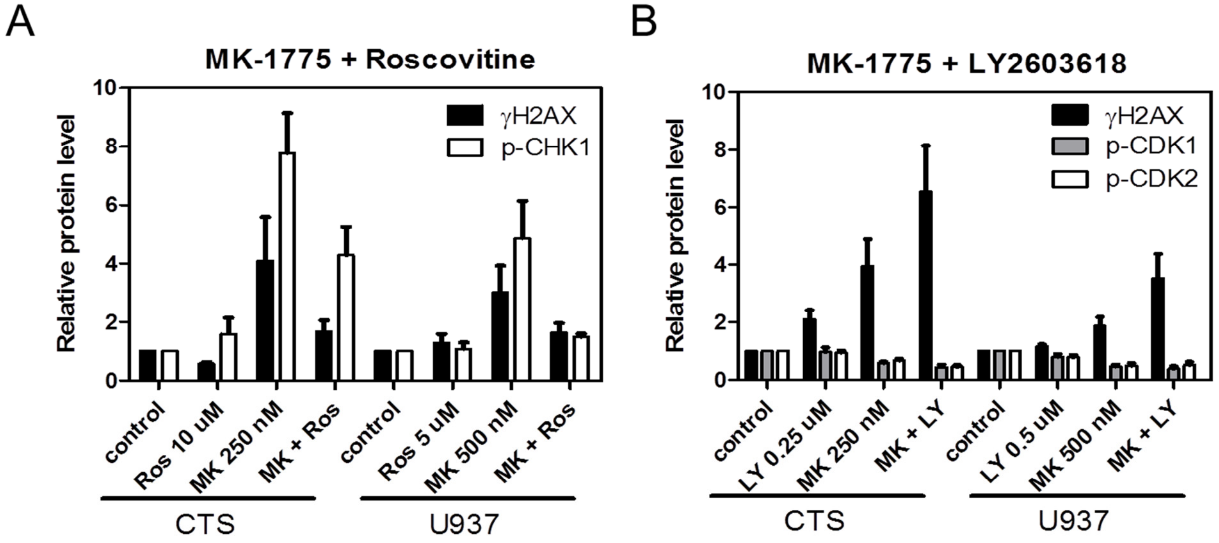


**Figure S3.** Densitometry measurements for the western blot experiments shown in Figures 4A and 5A. **Panel A:** Densitometry measurements for γH2AX and p-CHK1 expression from the western blot experiments shown to Figure 4A and the corresponding replicates were measured using Odyssey V3.0, normalized to β-actin and graphed as fold change compared to the no drug control. **Panel B:** Densitometry measurements for γH2AX, p-CDK1, and p-CDK2 expression from the western blot experiments shown in Figure 5A and the corresponding replicates were measured using Odyssey V3.0, normalized to β-actin and graphed as fold change compared to the no drug control. Western blot experiments were repeated at least three independent times.

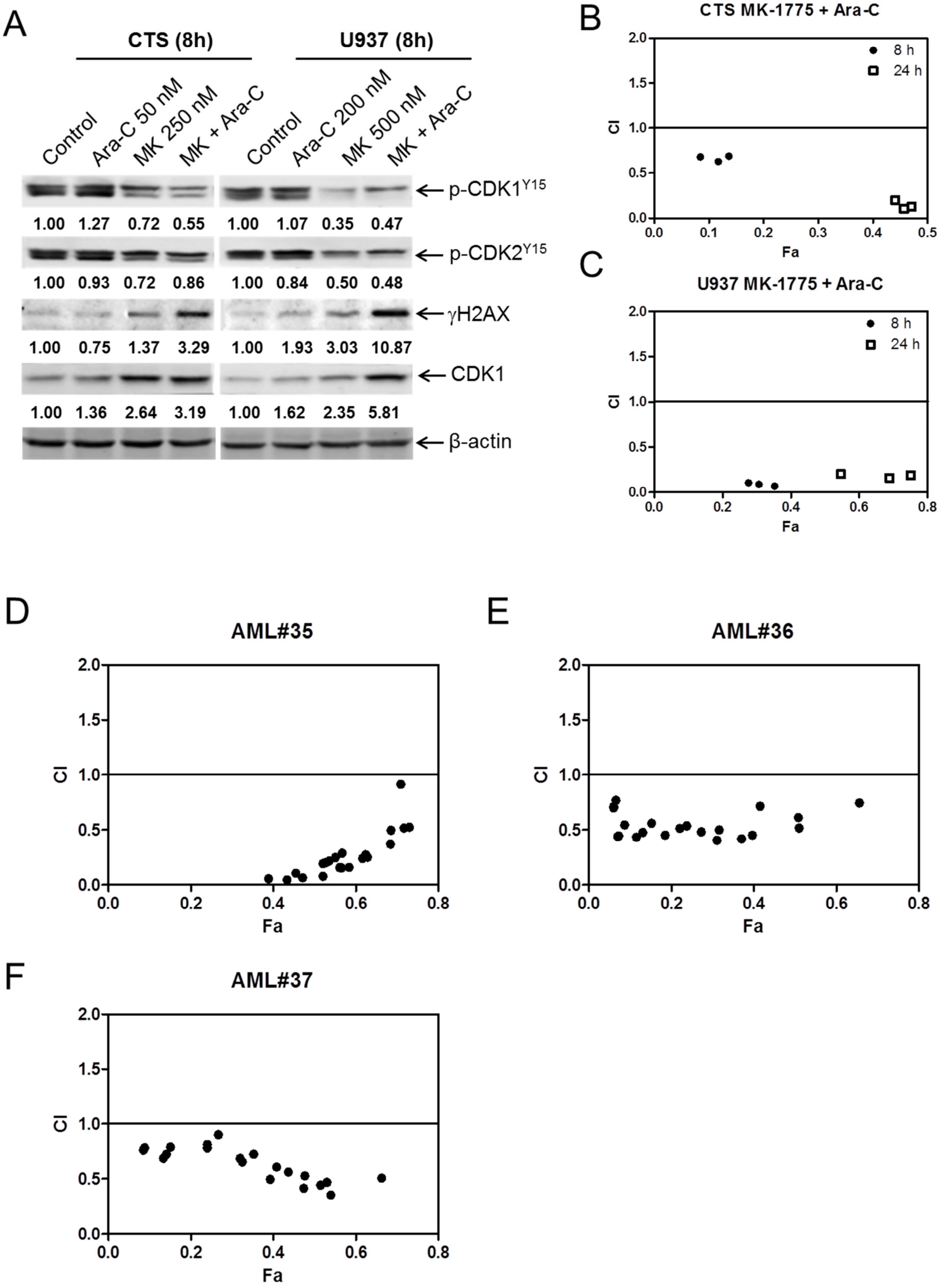
A
CTS (8h)
U937 (8h)
Control
Ara-C 50 nM
MK 250 nM
MK + Ara-C
Control
Ara-C 200 nM
MK 500 nM
MK + Ara-C
p-CDK1Y15
1.00 1.27 0.72 0.55 1.00 1.07 0.35 0.47
p-CDK2Y15
1.00 0.93 0.72 0.86 1.00 0.84 0.50 0.48
γH2AX
1.00 0.75 1.37 3.29 1.00 1.93 3.03 10.87
CDK1
1.00 1.36 2.64 3.19 1.00 1.62 2.35 5.81
β-actin
B
CTS MK-1775 + Ara-C
8 h
24 h
CI
Fa
C
U937 MK-1775 + Ara-C
8 h
24 h
CI
Fa
D
AML#35
CI
Fa
E
AML#36
CI
Fa
F
AML#37
CI
Fa

**Figure S4.** MK-1775 synergizes with cytarabine in AML cell lines and patient samples. **Panel A:** CTS and U937 cells were treated for 8 h. Whole cell lysates were subjected to Western blotting and probed with anti-p-CDK1, -p-CDK2, -γH2AX, -CDK1, -pCHK1 or -β-actin antibody. **Panels B and C:** CTS and U937 cells were treated with MK-1775 and cytarabine (Ara-C), alone or in combination, for 8 h or 24 h, then apoptotic events were determined by annexin V/PI staining and flow cytometry analyses. The CI and Fa values (combination index vs. fraction affected) were determined using CompuSyn Software and presented as CI vs. Fa plots. **Panels D-F:** Anti-leukemic interactions between MK-1775 and cytarabine were determined by MTT assays using primary patient samples. The data are presented as CI vs Fa plots. The CI and Fa values were determined using CompuSyn Software.
