# Supplementary material for: CHK1 plays a critical role in the anti-leukemic activity of the wee1 inhibitor MK-1775 in acute myeloid leukemia cells
Source: J Hematol Oncol. 2014 Aug 1;7:53. doi: 10.1186/s13045-014-0053-9 (PMC4237862; doi:10.1186/s13045-014-0053-9)
Supplement: Additional file 2: Table S1. — Cell cycle distribution of CTS and U937 cells following MK-1775 treatment. [file s13045-014-0053-9-S2.pdf]

Table S1. Cell cycle distribution of CTS and U937 cells after 500 nM MK-1775 treatment

		0 h	4 h	8 h	12 h	24 h	36 h	48 h
**CTS**	**Sub-G1**	4.92 ± 0.34	7.07 ± 0.06	19.03 ± 0.33	21.9 ± 0.15	51.00 ± 0.21	71.53 ± 0.35	84.73 ± 0.13
	**G1**	63.73 ± 0.03	58.00 ± 0.61	57.6 ± 0.17	52.63 ± 0.29	30.43 ± 0.18	17.23 ± 0.23	9.47 ± 0.27
	**S**	22.07 ± 0.07	25.97 ± 0.38	20.80 ± 0.25	23.87 ± 0.23	17.93 ± 0.18	10.87 ± 0.17	5.55 ± 0.18
	**G2/M**	9.32 ± 0.29	8.95 ± 0.28	2.58 ± 0.14	1.62 ± 0.01	0.67 ± 0.02	0.42 ± 0.04	0.25 ± 0.02
**U937**	**Sub-G1**	5.32 ± 0.31	5.05 ± 0.04	27.97 ± 0.29	30.8 ± 0.25	30.77 ± 0.72	38.17 ± 0.07	44.43 ± 0.26
	**G1**	65.63 ± 0.26	62.63 ± 0.35	50.77 ± 0.38	46.6 ± 0.15	35.83 ± 1.05	33.33 ± 0.73	33.4 ± 0.32
	**S**	19.70 ± 0.69	22.63 ± 0.22	16.7 ± 0.27	18.83 ± 0.29	28.2 ± 1.29	25.5 ± 0.60	19.43 ± 0.18
	**G2/M**	9.01 ± 0.13	9.67 ± 0.22	4.56 ± 0.27	3.76 ± 0.12	5.19 ± 0.43	2.97 ± 0.24	2.74 ± 0.16

The data are presented as means of triplicates ± standard errors from one representative experiment.
